# Arrhythmia and Death Following Percutaneous Revascularization in Ischemic Left Ventricular Dysfunction: Prespecified Analyses From the REVIVED-BCIS2 Trial

**DOI:** 10.1161/CIRCULATIONAHA.123.065300

**Published:** 2023-08-09

**Authors:** Divaka Perera, Holly P. Morgan, Matthew Ryan, Matthew Dodd, Tim Clayton, Peter D. O’Kane, John P. Greenwood, Simon J. Walsh, Roshan Weerackody, Adam McDiarmid, George Amin-Youssef, Julian Strange, Bhavik Modi, Timothy Lockie, Kai Hogrefe, Fozia Z. Ahmed, Miles Behan, Nicholas Jenkins, Eltigani Abdelaal, Michelle Anderson, Stuart Watkins, Richard Evans, Christopher A. Rinaldi, Mark C. Petrie

**Affiliations:** National Institute for Health Research Biomedical Research Center and British Heart Foundation Center of Research Excellence at the School of Cardiovascular Medicine and Sciences, King’s College London, United Kingdom; Guy’s and St Thomas’ NHS Foundation Trust, London, United Kingdom; National Institute for Health Research Biomedical Research Center and British Heart Foundation Center of Research Excellence at the School of Cardiovascular Medicine and Sciences, King’s College London, United Kingdom; National Institute for Health Research Biomedical Research Center and British Heart Foundation Center of Research Excellence at the School of Cardiovascular Medicine and Sciences, King’s College London, United Kingdom; London School of Hygiene & Tropical Medicine, United Kingdom; London School of Hygiene & Tropical Medicine, United Kingdom; Royal Bournemouth and Christchurch Hospital, Bournemouth, United Kingdom; Leeds Teaching Hospitals NHS Trust and University of Leeds, United Kingdom; Belfast Health and Social Care NHS Trust, United Kingdom; Barts Health NHS Trust, London, United Kingdom; Newcastle Hospitals NHS Foundation Trust, United Kingdom; King’s College Hospital NHS Foundation Trust, London, United Kingdom; University Hospitals Bristol NHS Foundation Trust, United Kingdom; University Hospitals of Leicester NHS Trust, United Kingdom; Royal Free Hospital, London, United Kingdom; Kettering General Hospital, Northampton, United Kingdom; Manchester Royal Infirmary, University NHS Foundation Trust, United Kingdom; Edinburgh Royal Infirmary, United Kingdom; Sunderland Royal Hospital, United Kingdom; Wythenshawe Hospital, Manchester, United Kingdom; Newcastle Hospitals NHS Foundation Trust, United Kingdom; Institute of Cardiovascular and Medical Sciences, University of Glasgow, United Kingdom; London School of Hygiene & Tropical Medicine, United Kingdom; Guy’s and St Thomas’ NHS Foundation Trust, London, United Kingdom; Institute of Cardiovascular and Medical Sciences, University of Glasgow, United Kingdom

**Keywords:** arrhythmias, cardiac, cardiomyopathies, death, sudden, cardiac, defibrillators, implantable, heart failure, myocardial revascularization, percutaneous coronary intervention

## Abstract

**Background:**

Ventricular arrhythmia is an important cause of mortality in patients with ischemic left ventricular dysfunction. Revascularization with coronary artery bypass graft or percutaneous coronary intervention is often recommended for these patients before implantation of a cardiac defibrillator because it is assumed that this may reduce the incidence of fatal and potentially fatal ventricular arrhythmias, although this premise has not been evaluated in a randomized trial to date.

**Methods:**

Patients with severe left ventricular dysfunction, extensive coronary disease, and viable myocardium were randomly assigned to receive either percutaneous coronary intervention (PCI) plus optimal medical and device therapy (OMT) or OMT alone. The composite primary outcome was all-cause death or aborted sudden death (defined as an appropriate implantable cardioverter defibrillator therapy or a resuscitated cardiac arrest) at a minimum of 24 months, analyzed as time to first event on an intention-to-treat basis. Secondary outcomes included cardiovascular death or aborted sudden death, appropriate implantable cardioverter defibrillator (ICD) therapy or sustained ventricular arrhythmia, and number of appropriate ICD therapies.

**Results:**

Between August 28, 2013, and March 19, 2020, 700 patients were enrolled across 40 centers in the United Kingdom. A total of 347 patients were assigned to the PCI+OMT group and 353 to the OMT alone group. The mean age of participants was 69 years; 88% were male; 56% had hypertension; 41% had diabetes; and 53% had a clinical history of myocardial infarction. The median left ventricular ejection fraction was 28%; 53.1% had an implantable defibrillator inserted before randomization or during follow-up. All-cause death or aborted sudden death occurred in 144 patients (41.6%) in the PCI group and 142 patients (40.2%) in the OMT group (hazard ratio, 1.03 [95% CI, 0.82–1.30]; *P*=0.80). There was no between-group difference in the occurrence of any of the secondary outcomes.

**Conclusions:**

PCI was not associated with a reduction in all-cause mortality or aborted sudden death. In patients with ischemic cardiomyopathy, PCI is not beneficial solely for the purpose of reducing potentially fatal ventricular arrhythmias.

**Registration:**

URL: https://www.clinicaltrials.gov; Unique identifier: NCT01920048.

Ischemic left ventricular systolic dysfunction is associated with significant morbidity and mortality. The leading cause of death in this population is sudden cardiac death, resulting largely from ventricular arrhythmias.^1,2^ Implantable cardioverter-defibrillators (ICDs) are widely used to mitigate the risk of sudden death in this population with primary prevention ICDs indicated in patients with a left ventricular ejection fraction <35%.^3^

The generation of ventricular arrhythmias in this population is believed to relate to both myocardial scarring and inducible ischemia.^1,4^ Treating the latter with coronary revascularization (with coronary artery bypass graft [CABG] or percutaneous coronary intervention [PCI]) is assumed to lead to improvement in left ventricular ejection fraction, which underlies contemporary guidelines that recommend deferral of ICD implantation for up to 90 days after revascularization in a primary prevention setting.^5,6^ Despite widespread clinical adoption, no high-quality data exist to support this approach. The recently published REVIVED-BCIS2 trial (Revascularisation for Ischaemic Ventricular Dysfunction–British Cardiovascular Intervention Society 2 Trial) found that revascularization with PCI did not reduce the incidence of all-cause death or hospitalization for heart failure in patients with ischemic left ventricular dysfunction.^7^ Among the subgroup who had an ICD or cardiac resynchronization therapy (CRT) defibrillator (CRT-D) implanted before randomization or within the first 90 days, those assigned to have PCI had fewer appropriate ICD therapies than those assigned to optimal medical therapy (OMT) alone, but this difference failed to reach statistical significance at 2 years. We now report the incidence of appropriate ICD therapies, resuscitated cardiac arrest, sustained ventricular arrhythmias, and death over the entire duration of follow-up in the PCI group compared with the group assigned to OMT alone.

## Methods

### Study Design

The trial design and primary outcome results have been reported previously.^7,8^ In brief, REVIVED-BCIS2 was a prospective, randomized, multicenter open-label trial in which patients with ischemic left ventricular systolic dysfunction were randomized to receive either PCI plus OMT or OMT alone. Participants were recruited from 40 hospitals in the United Kingdom. The trial protocol was registered before enrollment of the first patient (URL: https://www.clinicaltrials.gov. Unique identifier: NCT01920048; URL: https://www.isrctn.com. Unique identifier: ISRCTN 45979711) and was approved by the UK Health Research Authority. All patients provided written informed consent.

All participants enrolled in the REVIVED-BCIS2 trial were included in the present analyses. Patients were eligible for inclusion in the trial if they had ischemic left ventricular dysfunction, defined by the presence of severe left ventricular systolic dysfunction (left ventricular ejection fraction ≤35%), extensive coronary disease (British Cardiovascular Intervention Society Jeopardy Score ≥6), and evidence of viability in at least 4 dysfunctional myocardial segments that were amenable to revascularization with PCI. Those with myocardial infarction in the 4 weeks before randomization, sustained ventricular arrhythmia (ventricular tachycardia, ventricular fibrillation, or appropriate ICD discharges) in the 72 hours before randomization, or acutely decompensated heart failure were excluded.

The statistical analysis plan for this arrhythmia substudy was finalized before unblinding of trial group data from the arrhythmia case report form and is included in the Supplemental Material. The data that support the findings of this study and the analytic methods will be made available 1 year from completion of the trial on reasonable request to the corresponding author.

### Randomization and Data Collection

Participants were randomized in a 1:1 manner to either PCI plus OMT (PCI group) or OMT alone (OMT group). Randomization was stratified by center using randomly permuted blocks of varying size. Those randomized to the PCI group had revascularization attempted on all proximally diseased vessels subtending viable myocardium. All patients were initiated on OMT before enrollment, with subsequent uptitration of therapy supervised by a heart failure specialist at each recruiting site. Implantation of ICDs (and/or CRT) was an integral component of OMT in all patients, but the decision to implant a device was at the discretion of heart teams at recruiting centers. Device programming was carried out by implanting centers as per usual clinical care.

Clinical follow-up was carried out for a minimum of 24 months, with the last follow-up visit conducted within 3 months of the end of the overall trial follow-up period. Transthoracic echocardiography was performed at 6 and 12 months and analyzed by an echocardiography core laboratory at Guy’s and St Thomas’ NHS Foundation Trust, with readers blinded to treatment allocation and the temporal sequence of echocardiograms. Data relating to indication and timing of ICD and CRT insertion, aborted sudden death, and sustained ventricular arrythmias were collected from a dedicated arrhythmia case report form after completion of main trial follow-up using physical and electronic health records at the participating centers, including all scheduled and unscheduled device checks. ICD therapies were classified as appropriate or inappropriate by recruiting centers, as documented in the clinical device interrogation reports; these are the reports used for clinical and governance purposes in the National Health Service of the United Kingdom.

### Outcomes

The primary outcome was a composite of all-cause death or aborted sudden death (in turn, defined as an appropriate ICD therapy or a resuscitated cardiac arrest). Secondary outcomes included cardiovascular death or aborted sudden death, appropriate ICD therapy or sustained ventricular arrhythmia (any ventricular fibrillation or ventricular tachycardia >100 beats per minute that lasts for >30 seconds or requires termination in <30 seconds as a result of hemodynamic compromise^9^), and total number of appropriate ICD therapies (classified as none, 1, or ≥2 therapies). Outcomes were assessed in all patients over a minimum follow-up period of 24 months, except when sustained ventricular arrhythmia was a component, when it was restricted to patients who had an ICD or CRT device in situ, either at randomization or inserted during trial follow-up. The cause of death was as adjudicated by an independent blinded clinical events committee; ICD-related outcomes were site reported. All outcomes are defined in Table S1.

Analyses of the primary outcome were performed in these prespecified subgroups: device type (no device, CRT, non-CRT) indication for ICD (primary or secondary prevention), baseline left ventricular function, improvement in left ventricular function from baseline to 6 months (stratified by median change), extent of coronary artery disease, New York Heart Association functional class, and the type of recruiting center (dichotomized as centers that frequently/infrequently implanted ICDs or CRT devices in trial patients).

### Statistical Methods

Because this is a secondary analysis of a trial that has already been completed, a power calculation was not undertaken a priori. For illustration, if 40% of participants experience a primary outcome event over the entire duration of follow-up, equating to ≈250 events, the study would have at least 90% power to detect a hazard ratio of 0.65 or at least 80% power to detect a hazard ratio of 0.70, at a significance level of 5%.^10^

All analyses were performed on an intention-to-treat basis according to randomized assignment unless otherwise specified. The treatment groups were compared with a Cox proportional hazards model to calculate hazard ratios and 95% CIs. Unadjusted analyses were performed on the primary outcome, with time to first event measured from randomization to the date of death, withdrawal from the trial, or final trial follow-up. Adjusted analyses were also performed to account for variable timing of ICD or CRT implantation from randomization by including implantation as a time-varying covariate. Sensitivity analyses were performed on the primary outcome in the prespecified subgroup of patients who had an ICD or CRT device at randomization or received one within the first 90 days.

The *P* value for the treatment difference was calculated with a likelihood ratio test. The proportionality assumption underlying the Cox model was assessed with Nelson-Aalen plots by treatment group. Cumulative event rates were calculated and presented with Kaplan-Meier time-to-event curves. All analyses were undertaken with STATA software, version 17 (Stata Corp). Categorical demographic data are presented as counts (percentages); continuous data are presented as means (SDs) or medians (interquartile ranges), depending on the normality of distribution.

## Results

**Figure 1:**
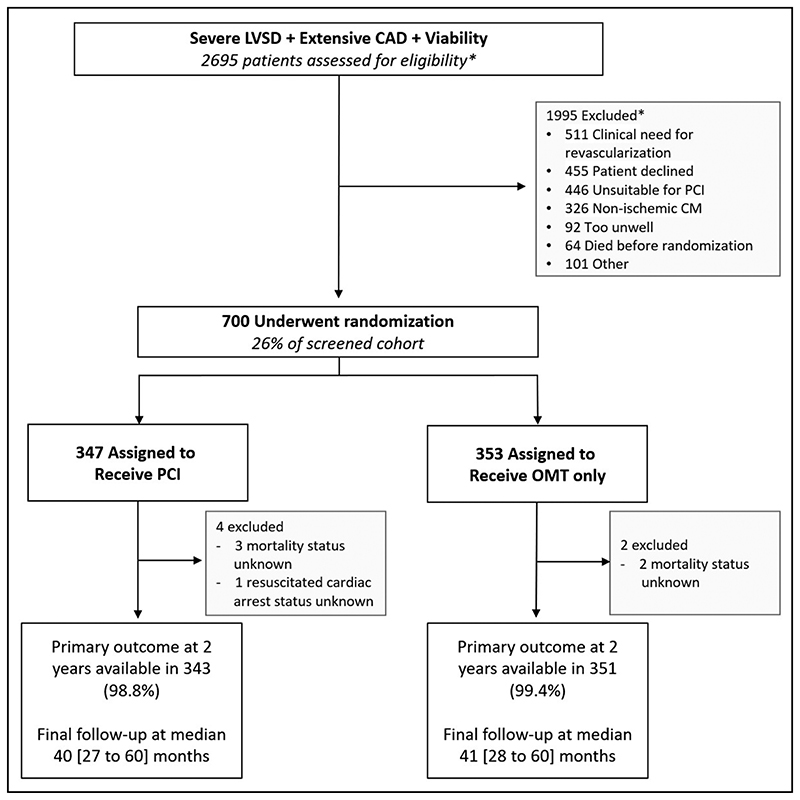
CONSORT diagram. CAD indicates coronary artery disease; CONSORT, Consolidated Standards of Reporting Trials; CM, cardiomyopathy; LVSD, left ventricular systolic dysfunction; OMT, optimal medical therapy; and PCI, percutaneous coronary intervention. *Number screened and numbers excluded have been extrapolated from interval screening logs that were conducted during the trial at multiple centers.

Between August 28, 2013, and March 19, 2020, 700 participants were enrolled; 347 were assigned to the PCI group and 353 to the OMT group (Figure 1). Primary outcome data at 2 years were available for 694 participants, with a median follow-up duration of 41 (27–60) months. Among the patients assigned to the PCI group, 334 (96.3%) underwent PCI at a median of 35 (15–57) days after randomization. In the OMT arm, 10.5% of patients underwent clinical events committee–adjudicated unplanned revascularization compared with 2.9% in the PCI arm. Detailed descriptions of the baseline assessments of left ventricular function and viability, titration of OMT, extent of coronary artery disease, and PCI procedural success have been reported previously.^7^ Baseline clinical and demographic characteristics were evenly distributed between the groups (Table 1).

### Primary Outcome

**Figure 2:**
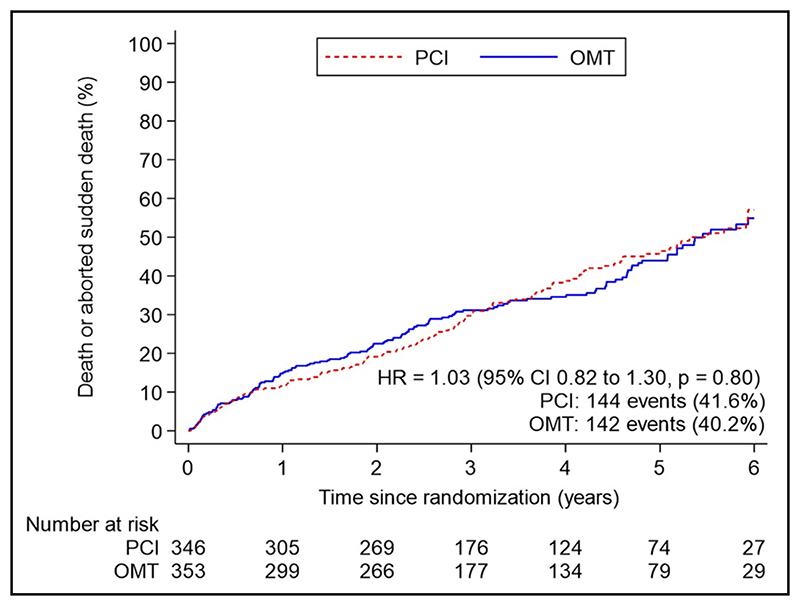
Primary outcome of all-cause death or aborted sudden death over all follow-up. Kaplan-Meier estimates of the cumulative incidence of death from any cause or aborted sudden death in a time-to-first event analysis. Incidence includes total events over the entire follow-up period in each group in the intention-to-treat population, censored at death, withdrawal from the trial, or date of final follow-up encounter. HR indicates hazard ratio; OMT, optimal medical therapy; and PCI, percutaneous coronary intervention.

**Figure 3:**
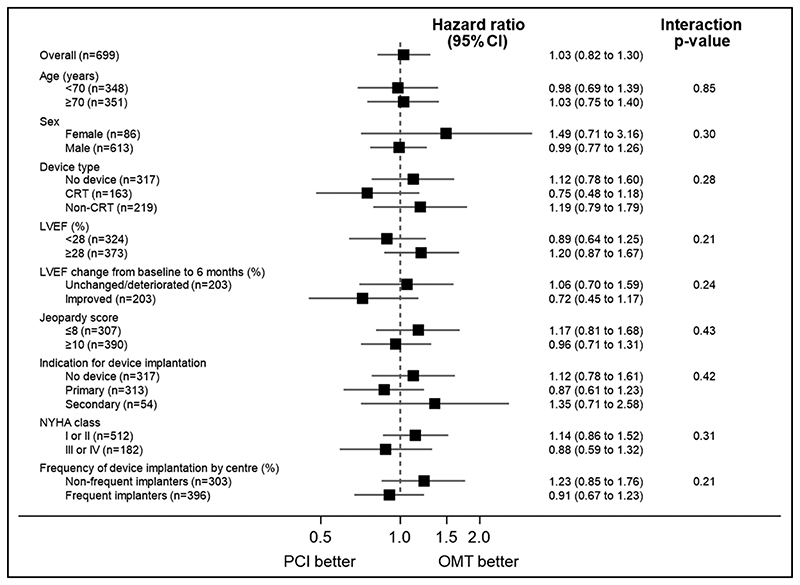
Primary outcome by prespecified subgroups. Forest plot showing hazard ratios for the primary outcome according to prespecified subgroups. Dashed line represents the null hypothesis of no treatment effect. The British Cardiovascular Intervention Society Jeopardy Score ranges from 0 to 12, with higher scores indicating a greater volume of myocardium subtended by diseased arteries. Implantable cardioverter defibrillator (ICD) indication (primary or secondary) includes both ICD and cardiac resynchronization therapy defibrillator implantations. Left ventricular ejection fraction (LVEF) change is from baseline to 6 months from core laboratory analyses; improved LVEF was defined as an improvement above the median change (>4.2%); unchanged or deteriorated LVEF was defined as below the median (≤4.2%). Recruiting center type defined by frequency of device implantation in the REVIVED-BCIS2 trial (Revascularisation for Ischaemic Ventricular Dysfunction): frequent if >50% of patients received a device, and infrequent if ≤50% received a device. NYHA indicates New York Heart Association heart failure class; OMT, optimal medical therapy; and PCI, percutaneous coronary intervention.

The primary outcome of all-cause death or aborted sudden death occurred in 144 patients (41.6%) in the PCI group and 142 patients (40.2%) within the OMT group (unadjusted hazard ratio, 1.03 [95% CI, 0.82–1.30]; *P*=0.80); adjusted hazard ratio, 1.02 [95% CI, 0.81–1.29]; *P*=0.84; Figure 2). There were 110 deaths (31.8%) in the PCI group and 115 (32.6%) in the OMT group. Aborted sudden death occurred in 44 patients (12.7%) in the PCI group and 47 patients (13.3%) in the OMT group (Table 2). The treatment effect for the primary outcome was consistent across all prespecified subgroups (Figure 3).

### Secondary Outcomes

The secondary outcome of cardiovascular death or aborted sudden death occurred in 111 of 346 patients in the PCI group and 120 of 353 in the OMT group (32.1% versus 34.0%; hazard ratio, 0.94 [95% CI, 0.73–1.22]). The composite of appropriate therapy or sustained ventricular arrhythmia occurred in 47 of 174 patients in the PCI group (27.0%) and 56 of 197 patients in the OMT group (28.4%; hazard ratio, 0.87 [95% CI, 0.59–1.28]; *P*=0.47). There was no between-group difference in the total number of appropriate ICD therapies (odds ratio, 1.11 [95% CI, 0.68–1.80]; *P*=0.68; Table 2).

**Figure 4:**
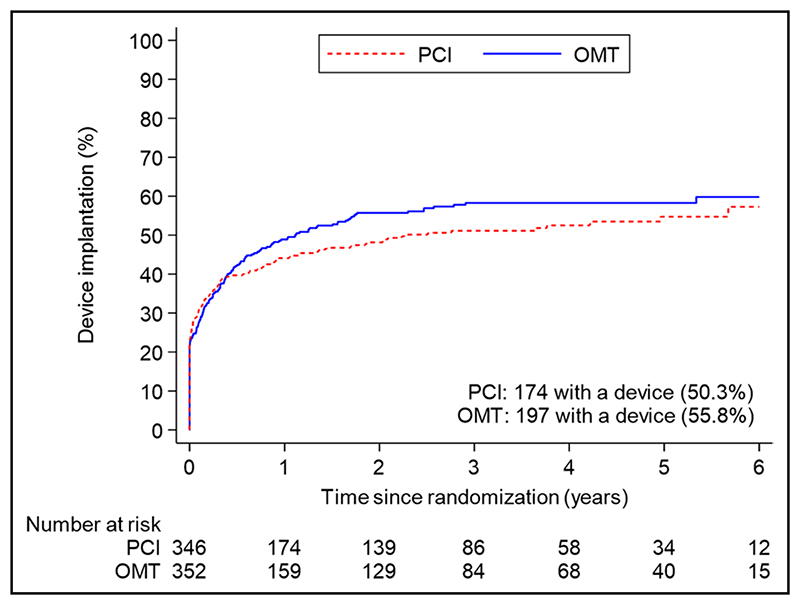
Cumulative device implantation by treatment group. The proportional hazards assumption seems to be violated (demonstrated by early separation of the curves, followed by gradual convergence over time); hence, a hazard ratio has not been calculated. OMT indicates optimal medical therapy; and PCI, percutaneous coronary intervention.

A total of 371 patients (53.1%) had an ICD or CRT device inserted before randomization or during followup: 174 of 347 (50.3%) in the PCI group and 197 of 353 (55.8%) in the OMT group (difference, −5.5% [95% CI, −12.9% to 1.9%]; Figure 4 and Table S2). The sensitivity analyses restricted to patients who had an ICD or CRT device in situ at randomization or received one within 90 days yielded results similar to those of the main analyses for the primary outcome (58 [47.2%] versus 62 [50.8%]; hazard ratio, 0.82 [95% CI, 0.57–1.18]; *P*=0.29) and the secondary outcomes (Tables S3 and S4).

## Discussion

In this analysis of the REVIVED-BCIS2 trial, a strategy of PCI in addition to OMT was not associated with a reduction in all-cause mortality or aborted sudden death in patients with ischemic left ventricular dysfunction. Furthermore, PCI was not associated with a reduction in the incidence of sustained ventricular arrhythmias or appropriate ICD therapies. The results suggest that patients with stable ischemic left ventricular dysfunction who are on guideline-directed medical therapy should not undergo PCI with the sole aim of reducing potentially fatal arrhythmias. The findings also challenge the widespread practice of undertaking CABG or PCI in most patients who are candidates for ICD implantation^11^ to reduce the arrhythmic risk, an effect believed be mediated by an improvement in left ventricular function. The absence of incremental improvement in left ventricular function in this cohort as a whole^7^ may also partly explain why PCI was not associated with a reduction in death or aborted sudden death in our study. The 2021 American College of Cardiology/American Heart Association guidelines recommend that revascularization should not be undertaken with the sole purpose of preventing recurrent sustained monomorphic ventricular tachycardia that is suspected to be related to scar.^6,12^ Our findings now extend this recommendation to the larger group of patients in whom ICD implantation is considered on primary prevention grounds.

In the preliminary report of the REVIVED-BCIS2 trial, appropriate ICD therapies tended to be less frequently observed at 2 years in patients assigned to have PCI, in the subgroup who had a device implanted at baseline or within the first 90 days.^7^ The current report extends arrhythmia follow-up of this subgroup and reveals that, when the entire duration of the trial is considered and competing mortality is accounted for, PCI is not associated with a reduction in aborted sudden death. This is consistent with the occurrence of the primary outcome in the entire trial cohort, which was not different between treatment arms. The recruited population was younger than the median age of patients newly diagnosed with heart failure in the United Kingdom^13^ but older than in other randomized trials in this field (mean ages: MADIT II [Multicenter Automatic Defibrillator Implantation 2 Trial], 64 years; MUSTT [Multicenter Unsustained Tachycardia Trial], 67 years; PROTECT-II [Prospective, Multicenter, Randomized Controlled Trial of the IMPELLA RECOVER LP 2.5 System Versus Intra Aortic Balloon Pump [IABP] in Patients Undergoing Non Emergent High Risk PCI], 68 years; DAPA HF [Dapagliflozin and Prevention of Adverse Outcomes in Heart Failure Trial], 66 years; CABG PATCH [Coronary Artery Bypass Graft Patch], 63 years; DANISH [Danish Study to Assess the Efficacy of ICDs in Patients With Nonischemic Systolic Heart Failure on Mortality], 63 years; STICH [Surgical Treatment for Ischemic Heart Failure Trial], 59 years; REVIVED-BCIS 2, 70 years).^14–20^

To the best of our knowledge, ours is the first trial to assess the effect of randomized allocation to PCI on arrhythmic risk in patients with stable ischemic left ventricular dysfunction. In the CABG PATCH trial, patients with ischemic left ventricular dysfunction and no history of ventricular arrhythmia who were undergoing CABG were randomized to receive either a surgically implanted ICD or no ICD; at an average follow-up of 32 months, there was no significant difference in all-cause mortality between groups.^18^ The authors hypothesized that the provision of CABG in both arms of the trial reduced the incidence of ventricular arrhythmias to the extent that the incremental benefit of ICD implantation was lost, but they were not able to confirm or refute this hypothesis because there was no medical therapy arm in that trial. In the STICH trial, which randomized patients with ischemic left ventricular dysfunction to either CABG or OMT, there was a significant reduction in the occurrence of sudden death in the CABG group.^21^ There was, however, a low rate of ICD or CRT implantation. Data on nonfatal ventricular arrhythmias were not captured, and the authors were unable to distinguish between sudden deaths due to ventricular arrhythmia from those caused by acute myocardial infarction or other causes.^22,23^

Site-reported completeness of revascularization in the REVIVED-BCIS2 trial was high, which would be expected to appreciably reduce the burden of inducible ischemia, particularly because the protocol recommended revascularization of all diseased vessels subtending viable myocardium. In this context, the inability to reduce all-cause mortality or aborted sudden death with PCI may indicate that ischemia is less important in the genesis of ventricular arrhythmias than scar, although the relative importance of these 2 factors cannot be definitively discerned without information on the burden and distribution of each. Furthermore, we cannot discount the possibility that PCI may cause periprocedural infarction, which could partially offset the benefit of reducing ischemic burden.^24,25^ Core laboratory–reported angiographic data are pending and may also provide further insights.

Our findings support those of the ISCHEMIA (International Study of Comparative Health Effectiveness With Medical and Invasive Approaches) investigators, who found no difference in all-cause mortality between patients with documented ischemia who underwent revascularization and those treated with medical therapy alone, although that trial excluded patients with impaired left ventricular function.^26^

Our study has some limitations. Because more than half of all patients received an ICD or CRT device, the trial has provided important insights into the potential mechanism of death and aborted sudden death in these cases, however, we had less ability to capture nonfatal arrhythmias in patients without devices and may have underestimated the overall event rate. This may have reduced statistical power, but given that the proportions of patients with devices were similar in the 2 trial groups, our estimation of treatment effect is unlikely to have been affected. The number of events detected provides 85% power to detect or exclude a hazard ratio of 0.70, in line with our initial power calculation, although we cannot exclude a smaller treatment effect with PCI. Second, the decision to implant a device was at the discretion of treating physicians and was based on clinical factors and regional differences in policy. Although there was no significant difference in the overall rates of device implantation over the course of the trial, it should be noted that the timing of implantation was not identical in both groups (Figure 4), although analyses adjusted for the timing of implantation and sensitivity analyses of patients with devices in situ at randomization provided conclusions similar to those of the unadjusted analyses. Third, the a priori definition of aborted sudden death included antitachycardia pacing or shocks, but we cannot be certain that all ICD therapies were delivered to treat arrhythmias that would have had fatal consequences without such therapies.^27,28^ Fourth, the protocol did not mandate postmortem interrogation of ICDs; although this does not affect the primary outcome, it is possible that we may have underestimated the frequency of sustained ventricular arrhythmias in both groups. Last, the classification of appropriate and inappropriate ICD therapy was based on the diagnoses recorded by the clinical teams at recruiting centers, and it is possible that device technicians and electrophysiologists may have been aware of the treatment assignment, so we cannot exclude ascertainment bias.

## Conclusions

In patients with ischemic left ventricular dysfunction, a strategy of PCI plus OMT was not associated with a reduction in death or aborted sudden death compared with a strategy of OMT alone. Patients with stable ischemic cardiomyopathy should not undergo PCI solely to reduce the burden of arrhythmias, and in patients who are eligible for an ICD, implantation does not need to be deferred until revascularization has been performed.

## Supplementary Material

Supplementary data

## Figures and Tables

**Table 1:** Baseline Demographic and Clinical Characteristics of the Intention-to-Treat Population

	PCI (n=346)	OMT (n=353)
Age, mean (SD), y	70.0 (9.0)	68.8 (9.1)
Male sex, n (%)	301 (870)	312 (88.4)
Race and ethnicity, n (%)		
White	305 (88.2)	328 (92.9)
Asian	32 (9.2)	1 7 (4.8)
Black	3 (0.9)	3 (0.9)
Mixed, other, or not reported	6 (1.7)	5 (1.4)
Site-reported LVEF (%)		
Mean (SD)	270 (6.6)	270 (6.9)
Median (IQR)	28.0 (23-32)	29.0 (22-33)
Core laboratory LVEF (%)		
Mean (SD)	31.9 (10.1)	31.9 (9.6)
Median (IQR)	32.1 (23.9-38.7) (n=263)	32.0 (24.8-378) (n=276)
BCIS jeopardy score* median (IQR)	10 (8-12)	10 (8-12)
β-Blocker, n (%)	314 (90.8)	319 (90.4)
ACE inhibitor/ARB/ARNI, n (%)	304 (879)	315 (89.5)
Amiodarone, n (%)	19 (5.5)	15 (4.2)
MRA, n (%)	175 (50.7)	170 (48.4)
Type of device, n (%)		
No device	265 (76.6)	277 (78.5)
ICD	45 (13.0)	33 (9.3)
CRT-D	31 (9.0)	38 (10.8)
CRT-P	5 (1.4)	5 (1.4)
Secondary prevention ICD, n (%)	21/76 (276)	14/71 (19.7)
Atrial fibrillation, n (%)	54 (16.9)	60 (179)
Advanced CKD, n (%)	77 (22.5)	57 (16.4)
QRS duration,† median (IQR), ms	112 (100–136) (n=237)	112 (98–138) (n=236)

ACE indicates angiotensin-converting enzyme; ARB, angiotensin receptor blocker; ARNI, angiotensin receptor neprilysin inhibitor; BCIS, British Cardiovascular Intervention Society; CKD, chronic kidney disease (advanced CKD defined as an estimated glomerular filtration rate <45 mL-min^-1^-1.73 m^-2^ or being established on dialysis); CRT-D, cardiac resynchronization therapy defibrillator; CRT-P, cardiac resynchronization therapy pacemaker; ICD, implantable cardioverter-defibrillator; IQR, interquartile range; LVEF, left ventricular ejection fraction; MRA, mineralocorticoid receptor antagonist; OMT, optimal medical therapy; and PCI, percutaneous coronary intervention.

* The BCIS Jeopardy Score is a quantification of the extent of myocardial jeopardy relating to clinically significant coronary artery stenoses. The score ranges from 0 (no significant coronary disease) to 12 (disease jeopardizing the whole left ventricular myocardium).

† Baseline QRS taken from ECG at randomization. Paced ECGs excluded.

**Table 2:** Primary and Secondary Outcomes Over All Follow-Up

	PCI, n (%)	OMT, n (%)	Unadjusted hazard/ odds ratio (95% CI)	*P* value
All patients				
All-cause death or aborted sudden death	144 (41.6)	142 (40.2)	1.03 (0.82–1.30)*	0.80
All-cause death	110	115		
Aborted sudden death	44	47		
Appropriate therapy	41	43		
Resuscitated cardiac arrest	6	6		
Cardiovascular death or aborted sudden death	111 (32.1)	120 (34.0)	0.94 (0.73–1.22)*	0.64
Cardiovascular death	76	88		
Cohort with ICD, CRT-D or CRT-P				
Appropriate ICD therapy or sustained ventricular arrhythmia	47/174 (27.0)	56/197 (28.4)	0.87 (0.59–1.28)*	0.47
Appropriate ICD therapy	41	43		
Sustained ventricular arrhythmia	47	55		
Cohort with ICD or CRT-D				
Any therapy (appropriate and inappropriate)	47/164 (28.7)	44/188 (23.4)	1.31 (0.82–2.12)	0.26
No. of appropriate therapies			1.11 (0.68–1.80)†	0.68
0	123/164 (75.0)	145/188 (77.1)		
1	15/164 (9.1)	14/188 (7.4)		
≥2	26/164 (15.9)	29/188 (15.4)		

CRT-D indicates cardiac resynchronization therapy defibrillator; CRT-P, cardiac resynchronization therapy pacemaker; ICD, implantable cardioverter-defibrillator; OMT, optimal medical therapy; and PCI, percutaneous coronary intervention.

* When adjusted for device implantation as a time-varying covariate, the hazard ratio for the primary outcome was 1.02 (95% CI, 0.81–1.29; *P*=0.84); for cardiovascular death or aborted sudden death, 0.90 (95% CI, 0.70–1.16; *P*=0.41); and for appropriate ICD therapy or sustained ventricular arrhythmia, 0.83 (95% CI, 0.57–1.20; *P*=0.32).

† Treatment effect is unadjusted odds ratio from an ordinal logistic regression model.

## Clinical Perspective

### What Is New?

- The rate of death or aborted sudden death remains high in ischemic left ventricular dysfunction, despite the use of guideline-directed medical therapy.
- Percutaneous coronary intervention was not associated with lower all-cause mortality, aborted sudden death, or sustained ventricular arrhythmias in patients with ischemic left ventricular dysfunction compared with optimal medical therapy alone.

### What Are the Clinical Implications?

- Patients with ischemic left ventricular dysfunction should not undergo percutaneous coronary intervention with the sole aim of preventing the occurrence of ventricular arrhythmias.
- In stable patients who are treated with guideline-directed medical therapy and meet criteria for implantable cardioverter defibrillator implantation, there is no evidence to support delaying implantation merely to assess the effect of percutaneous coronary intervention because the latter was not found to improve left ventricular function.

## Nonstandard Abbreviations and Acronyms

CABG: coronary artery bypass graft
CABG PATCH: Coronary Artery Bypass Graft Patch
CRT: cardiac resynchronization therapy
CRT-D: cardiac resynchronization therapy defibrillator
DANISH: Danish Study to Assess the Efficacy of ICDs in Patients With Nonischemic Systolic Heart Failure on Mortality
DAPA HF: Dapagliflozin and Prevention of Adverse Outcomes in Heart Failure Trial
ICD: implantable cardioverter-defibrillator
ISCHEMIA: International Study of Comparative Health Effectiveness With Medical and Invasive Approaches
MADIT II: Multicenter Automatic Defibrillator Implantation 2 Trial
MUSTT: Multicenter Unsustained Tachycardia Trial
OMT: optimal medical therapy
PCI: percutaneous coronary intervention
PROTECT-II: Prospective, Multi-center, Randomized Controlled Trial of the IMPELLA RECOVER LP 2.5 System Versus Intra Aortic Balloon Pump [IABP] in Patients Undergoing Non Emergent High Risk PCI
REVIVED-BCIS2: Revascularisation for Ischaemic Ventricular Dysfunction-British Cardiovascular Intervention Society 2 Trial
STICH: Surgical Treatment for Ischemic Heart Failure Trial

## References

[1] Gräni C, Benz DC, Gupta S, Windecker S, Kwong RY (2020). Sudden cardiac death in ischemic heart disease: from imaging arrhythmogenic substrate to guiding therapies. JACC Cardiovasc Imaging.

[2] Ryan M, Morgan H, Petrie MC, Perera D (2021). Coronary revascularisation in patients with ischaemic cardiomyopathy. Heart.

[3] National Institute for Health and Care Excellence (2014). Implantable cardioverter defibrillators and cardiac resynchronisation therapy for arrhythmias and heart failure Technology appraisal guidance [TA314].

[4] Canty JM, Suzuki G, Banas MD, Verheyen F, Borgers M, Fallavollita JA (2004). Hibernating myocardium: chronically adapted to ischemia but vulnerable to sudden death. Circ Res.

[5] Russo AM, Stainback RF, Bailey SR, Epstein AE, Heidenreich PA, Jessup M, Kapa S, Kremers MS, Lindsay BD, Stevenson LW (2013). ACCF/HRS/AHA/ ASE/HFSA/SCAI/SCCT/SCMR 2013 appropriate use criteria for implantable cardioverter-defibrillators and cardiac resynchronization therapy: a report of the American College of Cardiology Foundation Appropriate Use Criteria Task Force, Heart Rhythm Society, American Heart Association, American Society of Echocardiography, Heart Failure Society of America, Society for Cardiovascular Angiography and Interventions, Society of Cardiovascular Computed Tomography, and Society for Cardiovascular Magnetic Resonance. Heart Rhythm.

[6] Al-Khatib SM, Stevenson WG, Ackerman MJ, Bryant WJ, Callans DJ, Curtis AB, Deal BJ, Dickfeld T, Field ME, Fonarow GC (2018). 2017 AHA/ACC/ HRS guideline for management of patients with ventricular arrhythmias and the prevention of sudden cardiac death: a report of the American College of Cardiology/American Heart Association Task Force on Clinical Practice Guidelines and the Heart Rhythm Society. Circulation.

[7] Perera D, Clayton T, O’Kane PD, Greenwood JP, Weerackody R, Ryan M, Morgan HP, Dodd M, Evans R, Canter R (2022). REVIVED-BCIS2 Investigators. Percutaneous revascularization for ischemic left ventricular dysfunction. N Engl J Med.

[8] Perera D, Clayton T, Petrie MC, Greenwood JP, O’Kane PD, Evans R, Sculpher M, Mcdonagh T, Gershlick A, de Belder M (2018). REVIVED Investigators. Percutaneous revascularization for ischemic ventricular dysfunction: rationale and design of the REVIVED-BCIS2 trial: percutaneous coronary intervention for ischemic cardiomyopathy. JACC Heart Fail.

[9] Buxton AE, Calkins H, Callans DJ, DiMarco JP, Fisher JD, Greene HL, Haines DE, Hayes DL, Heidenreich PA, Miller JM (2006). American College of Cardiology/American Heart Association Task Force on Clinical Data Standards (ACC/AHA/HRS Writing Committee to Develop Data Standards on Electrophysiology). ACC/AHA/HRS 2006 key data elements and definitions for electrophysiological studies and procedures: a report of the American College of Cardiology/American Heart Association Task Force on Clinical Data Standards (ACC/AHA/HRS Writing Committee to Develop Data Standards on Electrophysiology). Circulation.

[10] Pocock SJ, Clayton TC, Stone GW (2015). Design of major randomized trials: part 3 of a 4-part series on statistics for clinical trials. J Am Coll Cardiol.

[11] Neumann FJ, Sousa-Uva M, Ahlsson A, Alfonso F, Banning AP, Benedetto U, Byrne RA, Collet JP, Falk V, Head SJ (2019). ESC Scientific Document Group. 2018 ESC/EACTS guidelines on myocardial revascularization. Eur Heart J.

[12] Lawton JS, Tamis-Holland JE, Bangalore S, Bates ER, Beckie TM, Bischoff JM, Bittl JA, Cohen MG, DiMaio JM, Don CW (2022). 2021 ACC/AHA/ SCAI guideline for coronary artery revascularization: a report of the American College of Cardiology/American Heart Association Joint Committee on Clinical Practice Guidelines. Circulation.

[13] NICOR (2020). National Heart Failure Audit (NHFA): 2020 summary report (2018/19 Data).

[14] Moss AJ, Zareba W, Hall WJ, Klein H, Wilber DJ, Cannom DS, Daubert JP, Higgins SL, Brown MW, Andrews ML (2002). Multicenter Automatic Defibrillator Implantation Trial II Investigators. Prophylactic implantation of a defibrillator in patients with myocardial infarction and reduced ejection fraction. N Engl J Med.

[15] Buxton AE, Lee KL, Fisher JD, Josephson ME, Prystowsky EN, Hafley G (1999). A randomized study of the prevention of sudden death in patients with coronary artery disease: Multicenter Unsustained Tachycardia Trial investigators. N Engl J Med.

[16] O’Neill WW, Kleiman NS, Moses J, Henriques JP, Dixon S, Massaro J, Palacios I, Maini B, Mulukutla S, Dzavik V (2012). A prospective, randomized clinical trial of hemodynamic support with Impella 2.5 versus intra-aortic balloon pump in patients undergoing high-risk percutaneous coronary intervention: the PROTECT II study. Circulation.

[17] McMurray JJV, Solomon SD, Inzucchi SE, Kober L, Kosiborod MN, Martinez FA, Ponikowski P, Sabatine MS, Anand IS, Bёlohlâvek J (2019). DAPA-HF Trial Committees and Investigators. Dapagliflozin in patients with heart failure and reduced ejection fraction. N Engl J Med.

[18] Bigger JT (1997). Prophylactic use of implanted cardiac defibrillators in patients at high risk for ventricular arrhythmias after coronary-artery bypass graft surgery: Coronary Artery Bypass Graft (CABG) Patch Trial Investigators. N Engl J Med.

[19] Køber L, Thune JJ, Nielsen JC, Haarbo J, Videbæk L, Korup E, Jensen G, Hildebrandt P, Steffensen FH, Bruun NE (2016). DANISH Investigators. Defibrillator implantation in patients with nonischemic systolic heart failure. N Engl J Med.

[20] Velazquez EJ, Lee KL, Deja MA, Jain A, Sopko G, Marchenko A, Ali IS, Pohost G, Gradinac S, Abraham WT, STICH Investigators (2011). Coronaryartery bypass surgery in patients with left ventricular dysfunction. N Engl J Med.

[21] Carson P, Wertheimer J, Miller A, O’Connor CM, Pina IL, Selzman C, Sueta C, She L, Greene D, Lee KL (2013). STICH Investigators. The STICH trial (Surgical Treatment for Ischemic Heart Failure): mode-of-death results. JACC Heart Fail.

[22] Tomaselli GF, Zipes DP (2004). What causes sudden death in heart failure?. Circ Res.

[23] John RM, Tedrow UB, Koplan BA, Albert CM, Epstein LM, Sweeney MO, Miller AL, Michaud GF, Stevenson WG (2012). Ventricular arrhythmias and sudden cardiac death. Lancet.

[24] Cheong BY, Muthupillai R, Wilson JM, Sung A, Huber S, Amin S, Elayda MA, Lee VV, Flamm SD (2009). Prognostic significance of delayed-enhancement magnetic resonance imaging: survival of 857 patients with and without left ventricular dysfunction. Circulation.

[25] Ricciardi MJ, Wu E, Davidson CJ, Choi KM, Klocke FJ, Bonow RO, Judd RM, Kim RJ (2001). Visualization of discrete microinfarction after percutaneous coronary intervention associated with mild creatine kinase-MB elevation. Circulation.

[26] Hochman JS, Anthopolos R, Reynolds HR, Bangalore S, Xu Y, O’Brien SM, Mavromichalis S, Chang M, Contreras A, Rosenberg Y (2023). Survival after invasive or conservative management of stable coronary disease. Circulation.

[27] Moss AJ, Schuger C, Beck CA, Brown MW, Cannom DS, Daubert JP, Estes NA, Greenberg H, Hall WJ, Huang DT (2012). MADIT-RIT Trial Investigators. Reduction in inappropriate therapy and mortality through ICD programming. N Engl J Med.

[28] Schuger CD, Ando K, Cantillon DJ, Lambiase PD, Mont L, Joung BY, Peress D, Yong P, Wold N, Daubert JP (2021). Assessment of primary prevention patients receiving an ICD: systematic evaluation of ATP: APPRAISE ATP. Heart Rhythm O2.

