# Supplementary material for: Arrhythmia and Death Following Percutaneous Revascularization in Ischemic Left Ventricular Dysfunction: Prespecified Analyses From the REVIVED-BCIS2 Trial
Source: Circulation. Author manuscript; Available in PMC 2023 Sep 24. (PMC10487377; doi:10.1161/CIRCULATIONAHA.123.065300)
Supplement: Supplementary data [file EMS185033-supplement-Supplementary_data.pdf]

SUPPLEMENTAL MATERIAL

Arrhythmia and death following percutaneous revascularization in ischemic left ventricular dysfunction. Prespecified analyses from the REVIVED-BCIS2 trial.

Contents

REVIVED Sites and Investigators 2
Trial Organization and Oversight 5

Supplemental Tables

Table S1. Outcome Definitions 8
Table S2: Timing of device implantation........ 9
Table S3 – Sensitivity analysis: primary outcome........ 9
Table S4 – Sensitivity analysis: secondary outcomes 10

REVIVED Trial Arrhythmia Study Statistical Analysis Plan........ 11

REVIVED Sites and Investigators

This list is ordered by the number of patients enrolled by each center. There were many more individuals who made contributions to the REVIVED-BCIS2 Trial at each participating center but have not been named below; we are very grateful for their efforts.

Center	Principal Investigator	Co-Investigators	Coordinators
Guy's and<br>St Thomas' Hospital	Prof Divaka Perera	Prof Amedeo Chiribiri<br>Prof Gerry Carr-White<br>Dr Antonis Pavlidis<br>Prof Simon Redwood<br>Dr Brian Clapp<br>Prof Aldo Rinaldi<br>Dr Haseeb Rahman<br>Dr Natalia Briceno	Sophie Arnold<br>Amy Raynsford<br>Karen Wilson<br>Lucy Clack
Golden Jubilee National Hospital, Glasgow	Prof Mark Petrie	Dr Margaret McEntegart<br>Dr Stuart Watkins<br>Dr Aadil Shaukat<br>Dr Paul Rocchiccioli	Marion McAdam<br>Elizabeth McPherson<br>Louise Cowan<br>Marie Wood
Barts Heart Centre, London	Dr Roshan Weerackody	Dr Ceri Davies<br>Dr Elliot Smith<br>Dr Bhavik Modi	Bindu Mathew<br>Oliver Mitchelmore<br>Rita Adrego<br>Mervyn Andiapen
Royal Bournemouth Hospital	Dr Peter O'Kane	Dr Jehangir Din	Sarah Kennard<br>Sarah Orr<br>Cathie Purnell
Leeds General Infirmary	Prof John Greenwood	Dr Jonathan Blaxill<br>Dr Abdul Mozid	Michelle Anderson<br>Kathryn Somers
Royal Victoria Hospital, Belfast	Dr Lana Dixon	Dr Simon Walsh<br>Dr Mark Spence	Patricia Glover<br>Caroline Brown
Freeman Hospital, Newcastle	Dr Richard Edwards	Dr Adam McDiarmid<br>Dr Mohaned Egred	Alla Narytnyk<br>Vera Wealleans
King's College Hospital, London	Dr George Amin-Youssef	Prof Ajay Shah<br>Prof Theresa McDonagh<br>Dr Jonathan Byrne<br>Dr Nilesh Pareek	Jonathan Breeze<br>Catherine Antao
Bristol Royal Infirmary	Dr Kalpa De Silva	Dr Julian Strange<br>Dr Tom Johnson<br>Dr Angus Nightingale	Laura Gallego<br>Cristina Medina
Glenfield Hospital, Leicester	Prof Anthony Gershlick	Prof Gerald McCann<br>Dr Andrew Ladwiniec<br>Prof Iain Squire	Joanna Davison<br>Kris Kenmuir-Hogg
St George's Hospital, London	Prof James Spratt	Dr Claudia Cosgrove<br>Dr Rupert Williams<br>Dr Sam Firoozi<br>Dr Pitt Lim	Giovanna Bonato<br>Vennessa Sookhoo
Pinderfields Hospital, Wakefield	Dr Dwayne Conway	Dr Paul Brooksby	Judith Wright<br>Donna Exley
New Cross Hospital, Wolverhampton	Dr James Cotton	Dr Richard Horton	Stella Metherell<br>Andrew Smallwood

Center	Principal Investigator	Co-Investigators	Coordinators
Kettering General Hospital	Dr Kai Hogrefe	Dr Adrian Cheng	Charmaine Beirnes<br>Sian Sidgwick
Royal Free Hospital, London	Dr Tim Lockie	Dr Niket Patel<br>Dr Roby Rakhit	Nina Davies<br>Angelique Smit
Manchester Royal Infirmary	Dr Fozia Ahmed	Dr Cara Hendry<br>Dr Farzin Fath-Odoubadi<br>Dr Douglas Fraser<br>Dr Mamas Mamas	Anu Oommen<br>Thabitha Charles
Royal Infirmary of Edinburgh	Dr Miles Behan	Dr Alan Japp	Belinda Rif
Sunderland Royal Hospital	Dr Nicholas Jenkins	Dr Sam McClure	Pauline Oates<br>Karen Martin
Wythenshawe Hospital	Dr Eltigani Abdelaal	Dr Jaydeep Sarma<br>Dr Sanjay Shastri<br>Dr Jo Riley	Sarra Giannopoulou<br>Sophie Quinn
Liverpool Heart and Chest Hospital	Dr Pradeep Magapu	Prof Rod Stables<br>Dr David Wright	Janet Barton<br>Nichola Clarkson
Southampton General Hospital	Dr Michael Mahmoudi	Dr Andrew Flett<br>Prof Nick Curzen	Judith Radmore<br>Sam Gough
Royal Devon & Exeter Hospital	Dr Andrew Ludman	Dr Hibba Kurdi	Samantha Keenan
University Hospitals Coventry and Warwickshire	Prof Prithwish Banerjee	Dr Luke Tapp	Nigel Edwards<br>Catherine Gibson
Lister Hospital, Stevenage	Dr Neville Kukreja	Dr Mary Lynch	Claire Barratt
The James Cook University Hospital, Middlesbrough	Dr Mark de Belder	Dr Jeet Thambyrajah<br>Dr Neil Swanson	Cath Richardson<br>Bev Atkinson
Derriford Hospital, Plymouth	Dr Girish Viswanathan		Darren Waugh
Worcestershire Acute Hospitals	Dr Helen Routledge	Dr Jasper Trevelyan	Angela Doughty
Worthing Hospital	Dr Nick Pegge	Dr Sukhbir Dhamrait	Sally Moore
Blackpool Victoria Hospital	Dr Gavin Galasko	Dr Christopher Cassidy	Natalia Waddington
Dorset County Hospital	Dr Tim Edwards	Dr Javed Iqbal<br>Dr Fraser Witherow	Jenny Birch<br>Melanie Munro
Salisbury District Hospital	Dr Tim Wells	Dr Manas Sinha	Linda Frost
Birmingham Heartlands Hospital	Dr Kaeng Lee	Dr James Beattie<br>Dr Mike Pitt	Alan Chung
Great Western Hospital, Swindon	Dr Steve Ramcharitar		Laura McCafferty

Center	Principal Investigator	Co-Investigators	Coordinators
Ninewells Hospital, Dundee	Dr Thomas Martin	Dr John Irving<br>Dr Zaid Iskandar	Anita Hutcheon
Northern General Hospital, Sheffield	Dr Julian Gunn	Dr Abdallah Al-Mohammad	Michael Agyemang
Queen Alexandra Hospital, Portsmouth	Dr Huw Griffiths	Prof Paul Kalra	Serena Howe
Royal Oldham Hospital	Dr Tim Gray	Dr Jolanta Sobolewska	Louise Morby
Basingstoke and North Hampshire Hospital	Dr Jason Glover	Dr James Beynon	Janet Knight
North Wales Cardiac Centre	Dr Paul Das	Dr Chris Bellamy	Emily Harman
The York Hospital	Mr Maurice Pye	Dr Simon Megarry	Yvonne McGill<br>Heidi Redfearn

Trial Organization and Oversight

Trial Steering Committee

Prof Andrew Clark, Chair of Clinical Cardiology, Castle Hill Hospital, Hull (Chair)

Mrs Helen Williams, Pharmacist, NHS Southwark Clinical Commissioning Group, London

Dr Pablo Perel, Epidemiologist, London School of Hygiene & Tropical Medicine

Dr David Walker, Consultant Cardiologist, Conquest Hospital, St. Leonards-on-Sea

Prof Rod Stables, Consultant Cardiologist, Liverpool Heart and Chest Hospital

Prof Divaka Perera, Chief Investigator, King's College London

Ms Liz Bestic, Patient, Carer and Public representative

Mrs Paula Young, Patient, Carer and Public representative

Mrs Helen Datta, Patient, Carer and Public representative

Mr Jeremy Dearling, Patient, Carer and Public representative

Data and Safety Monitoring Committee

Dr Peter Ludman, Consultant Cardiologist, Queen Elizabeth Hospital, Birmingham (Chair)

Dr Suzanna Hardman, Consultant Cardiologist, The Whittington Hospital, London

Dr Louise Brown, Senior Statistician, MRC Clinical Trials Unit at University College London

Clinical Events Committee

Prof Roxy Senior, Professor of Cardiology, Royal Brompton Hospital, London (Chair)

Dr Zaheer Yousef, Consultant Cardiologist, University Hospital of Wales

Dr Rajan Sharma, Consultant Cardiologist, St George's Hospital, London

Dr Shazia Hussain, Interventional Cardiologist, University Hospitals of Leicester NHS Trust

Dr Stephen Hoole, Consultant Cardiologist, Royal Papworth Hospital

Dr Ninian Lang, Reader in Cardiology, University of Glasgow

Dr Kieran Docherty, Clinical Lecturer in Cardiology, University of Glasgow

Dr Roy Gardner, Consultant Cardiologist, Golden Jubilee National Hospital, Glasgow

Prof Andrew Sharp, Consultant Cardiologist, University Hospital of Wales

Dr Ricardo Petraco, Consultant Cardiologist, Imperial College Healthcare NHS Trust

Dr Vasileios Panoulas, Consultant Cardiologist, Royal Brompton and Harefield Hospitals

Dr Andreas Schuster, Consultant Cardiologist, Universitätsmedizin Göttingen, Germany

Dr Kaleab Asrress, Consultant Cardiologist, Bankstown-Lidcombe Hospital, Australia

Dr Matthew Lee, Clinical Lecturer in Cardiology, University of Glasgow

Prof Pardeep Jhund, Professor of Cardiology and Epidemiology, University of Glasgow

Dr Eugene Connolly, Director, Global Clinical Trial Partners, Glasgow

Prof Raj Kharbanda, Consultant Cardiologist, John Radcliffe Hospital, Oxford

Ms Farandeep Dhaliwal, London School of Hygiene & Tropical Medicine (Admin)

Project Management Group

Prof Divaka Perera, King's College London

Prof Tim Clayton, London School of Hygiene & Tropical Medicine

Mr Richard Evans, London School of Hygiene & Tropical Medicine

Ms Ruth Canter, London School of Hygiene & Tropical Medicine

Mr Steven Robertson, London School of Hygiene & Tropical Medicine

Mrs Sophie Arnold, Guy's and St Thomas' Hospital, London

Dr Bhavik Modi, King's College London

Dr Matthew Ryan, King's College London

Dr Holly Morgan, King's College London

Mrs Rosemary Knight, London School of Hygiene & Tropical Medicine

Miss Rebecca Matthews, London School of Hygiene & Tropical Medicine

Mrs Lucy Clack, Guy's and St Thomas' Hospital, London

Ms Josenir Astarci, London School of Hygiene & Tropical Medicine (Admin)

Medical Therapy Committee

Prof Michael Marber, Professor of Cardiology, King's College London

Prof Aldo Rinaldi, Consultant Cardiologist, Guy's and St Thomas' Hospital, London

Dr Stam Kapetanakis, Consultant Cardiologist, Guy's and St Thomas' Hospital, London

Prof Mark Petrie, Consultant Cardiologist, University of Glasgow

Prof Theresa McDonagh, Consultant Cardiologist, King's College Hospital, London

Trial Statisticians

Ms Joanne Dobson, London School of Hygiene & Tropical Medicine

Mr Matthew Dodd, London School of Hygiene & Tropical Medicine

Prof Tim Clayton, London School of Hygiene & Tropical Medicine

Core Laboratories

Dr Stam Kapetenakis (Echocardiography Lead), Guy's and St Thomas' Hospital, London

Prof Amedeo Chiribiri (Cardiovascular Magnetic Resonance Lead), King's College London

Dr Margaret McEntegart (Coronary Angiography Lead), University of Glasgow

Dr Holly Morgan, King's College London

Dr Matthew Ryan, King's College London

Dr Saad Ezad, King's College London

Echocardiography Core Laboratory Readers

Ms Jane Draper, Guy's and St Thomas' Hospital, London

Ms Sheila Subbiah, Guy's and St Thomas' Hospital, London

Ms Annabel Oraa, Guy's and St Thomas' Hospital, London

Ms Olga Khaleva, Guy's and St Thomas' Hospital, London

Dr Haotian Gu, Guy's and St Thomas' Hospital, London

Dr Sarah Blake, Guy's and St Thomas' Hospital, London

Ms Emily Denman, King's College Hospital, London

Ms Almira Whittaker, King's College Hospital, London

Ms Marilou Huang, King's College Hospital, London

Ms Sandya Nandakumar, King's College Hospital, London

Dr Joseph Okafor, Guy's and St Thomas' NHS Foundation Trust, London

Dr Oleksandr Danylenko, Guy's and St Thomas' NHS Foundation Trust, London

Table S1. Outcome Definitions

Outcome	Definition
Aborted sudden death	An appropriate ICD therapy or a resuscitated cardiac arrest
Appropriate ICD therapy	Anti-tachycardia pacing +/- defibrillation for ventricular tachycardia or ventricular fibrillation
Cardiovascular Death	All deaths where there is no clinical or post-mortem evidence of a non-cardiovascular etiology, as adjudicated by the independent Clinical Events Committee
Inappropriate therapy	Anti-tachycardia pacing +/- defibrillation delivered in the absence of a ventricular tachyarrhythmia
Multiple therapies	When multiple therapies have been delivered in in quick succession, any additional appropriate therapy that is >5 minutes after the preceding therapy will be regarded a separate episode of therapy. A ventricular arrhythmia storm is defined as >3 separate therapies within a 24 hours period.
Non-sustained ventricular arrhythmia	Any ventricular arrhythmia greater than 3 beats but less than 30 seconds, receiving no therapy
Secondary prevention	ICD placement in a patient with prior cardiac arrest, sustained VT, or syncope confirmed or suspected to be due to ventricular arrhythmia
Sustained ventricular arrhythmia	Ventricular tachycardia >100bpm that lasts for more than 30 seconds OR requires termination (e.g. cardioversion) in less than 30 seconds because of hemodynamic compromise OR any ventricular fibrillation

Table S2: Timing of device implantation

	PCI (n = 346)	OMT (n = 353)
Device implanted at any time	174 (50.2%)	197 (55.8%)
Device implanted pre-randomization	81 (23.4%)	76 (21.5%)
Device implanted post-randomization	93 (26.9%)	121 (34.3%)
Device type (implanted at any time)<br>ICD<br>CRT-D<br>CRT-P	 93 (26.9%)<br>71 (20.5%)<br>10 (2.9%)	 107 (30.3%)<br>81 (22.9%)<br>9 (2.5%)
Time from randomization to implantation (at any time) in days, median (IQR)	7 (-50 to 119)	38 (-38 to 187)
Time from implantation to randomization (pre-randomization implants) in days, median (IQR)	-62 (-172 to -19)	-53 (-198 to -25)
Time from randomization to implantation (post-randomization implants) in days, median (IQR)	109 (32 to 384)	139 (52 to 334)

Table S3 – Sensitivity analysis: primary outcome

Restricted to pre-specified cohort with an ICD and/or CRT in situ within 90 days of randomization

	PCI (n = 123)	OMT (n = 122)
All-cause death or aborted sudden death	58 (47.2%)	62 (50.8%)
All-cause death	34	44
Aborted sudden death	30	32
Appropriate therapy	30	32
Resuscitated cardiac arrest	1	0
Unadjusted hazard ratio (95% CI)	0.82 (0.57 to 1.18); p = 0.29	
Adjusted* hazard ratio (95% CI)	0.82 (0.57 to 1.17); p = 0.28	

** adjusted for device implantation as a time-varying covariate; CI = confidence interval.*

It should be noted that there is a slight difference in the number of patients comprising this subgroup in the NEJM publication, compared to the current report, as the latter uses the arrhythmia CRF and precise implant dates compared to the former, which used site reported data indexed to visit date.

Table S4 – Sensitivity analysis: secondary outcomes
Restricted to pre-specified cohort with an ICD and/or CRT in situ within 90 days of randomization

	PCI	OMT	Unadjusted hazard ratio (95% CI)	p-value
All patients				
CV death or aborted sudden death	46/123 (37.4)	55/122 (45.1)	0.74 (0.50 to 1.09)	0.13
CV death	21	33		
Appropriate ICD therapy	30	32		
Resuscitated cardiac arrest	1	0		
Cohort with ICD, CRT-D or CRT-P				
Appropriate ICD therapy or sustained VA	34/123 (27.6)	38/122 (31.1)	0.78 (0.49 to 1.25)	0.30
Appropriate ICD therapy	30	32		
Sustained VA	34	37		
Cohort with ICD or CRT-D				
Total number of appropriate therapies			0.88 (0.50 to 1.57) *	0.66
0	88/118 (74.6)	84/116 (72.4)		
1	10/118 (8.5)	9/116 (7.8)		
≥2	20/118 (16.9)	23/116 (19.8)		

** treatment effect is unadjusted odds ratio from an ordinal logistic regression model.*

DocuSign Envelope ID: B93A3251-5F44-42D1-8DF5-9078C5F17443

Guy's and St Thomas' NHS
NHS Foundation Trust

REVIVED

Statistical Analysis Plan

Arrhythmia Sub-study

Version 1.1

19th August 2022

DocuSign Envelope ID: B93A3251-5F44-42D1-8DF5-9078C5F17443

FINAL VERSION APPROVED BY:

Name	Signature	Date
DIVAKA PERERA	DocuSigned by: [signature] 99D449AE8FF9434...	8/19/2022
Tim Clayton	DocuSigned by: Tim Clayton 2116E431B414456...	8/20/2022

CURRENT VERSION APPROVED BY (IF CHANGED FROM FINAL):

Name	Signature	Date
DIVAKA PERERA	DocuSigned by: [signature] 99D449AE8FF9434...	8/19/2022
Tim Clayton	DocuSigned by: Tim Clayton 2116E431B414456...	8/20/2022

REVISION OF FINAL VERSION CHRONOLOGY			
**VERSION NUMBER**	**EFFECTIVE DAT**	**REASON FOR CHANGE**	**CHANGE APPROVED BY (NAME)**
**1.1**	**19/08/22**	**Additional of power calculation**	**Divaka Perera**

DocuSign Envelope ID: B93A3251-5F44-42D1-8DF5-9078C5F17443

Background

REVIVED-BCIS2 is a multi-centre randomised controlled trial investigating whether percutaneous coronary intervention (PCI) improves clinical outcomes in patients with ischaemic left ventricular systolic dysfunction (defined by the presence of a severely reduced left ventricular ejection fraction) extensive coronary artery disease and demonstrable myocardial viability, compared with optimal medical therapy (OMT) alone (1). The primary composite outcome of the trial is all-cause death and hospitalisation for heart failure (HHF).

The leading cause of death in this population is sudden cardiac death, largely resulting from ventricular arrhythmias (2, 3). To mitigate this, guidelines recommend the routine implantation of an implantable cardiac defibrillator (ICD) for patients with coronary disease and a left ventricular ejection fraction <35% (4). The addition of cardiac resynchronisation therapy (CRT) is recommended in patients with heart failure in sinus rhythm with an LV ejection fraction of <35% and QRS duration > 120 ms (4).

The main drivers of ventricular arrhythmia are often considered to be the presence of scar related to previous myocardial infarction (which forms a substrate for re-entrant circuits to form within the ventricular myocardium) and myocardial ischaemia (which prolongs repolarisation and may lead to ventricular ectopy which triggers a re-entrant circuit) (2, 5). Preventing the development of myocardial ischaemia by coronary revascularisation has the potential to reduce the burden of ventricular arrhythmias and sudden cardiac death in this population. In the CABG-PATCH trial, the prophylactic insertion of an ICD in patients with ischaemic LVSD who were undergoing coronary artery bypass grafting (CABG) did not reduce the risk of death over a median follow-up of 32 months, with the authors suggesting that the provision of coronary revascularisation (which was performed in all patients) had reduced the arrhythmic burden such that ICD implantation would not provide further prognostic benefit (6). In the STICH trial, comparing CABG to OMT in patients with ischaemic left ventricular systolic dysfunction, the rate of sudden death was lower in patients receiving CABG, with the benefit being particularly apparent in patients surviving more than 2 years after randomisation (7). Neither of these trials, however, specifically addressed the question of reduction in ventricular arrhythmias and sudden death. Current clinical guidelines for the prevention of sudden cardiac death recommend coronary revascularisation prior to implantation of an implantable cardioverter defibrillator without strong supporting evidence (8).

The REVIVED-BCIS2 trial provides a unique opportunity to study the effect of coronary revascularisation with PCI on the occurrence of ventricular arrhythmias and sudden cardiac death within a randomised trial. It is estimated that more than 50% of patients in REVIVED-BCIS2 will have a cardiac implantable electronic device (CIED) in situ, either implanted before or during the trial follow up.

The aim of this sub-study is therefore to capture granular and longer-term data on arrhythmic burden within this cohort. We will collect data from all implanted CIEDs, including pacemakers and loop recorders, which allows robust capture of fatal and non-fatal ventricular arrhythmia episodes.

DocuSign Envelope ID: B93A3251-5F44-42D1-8DF5-9078C5F17443

Study Objectives

- To determine whether a strategy of revascularisation by PCI alters incidence of death or aborted sudden death, compared to OMT alone
- To determine whether a strategy of revascularisation by PCI reduces the frequency of appropriate ICD therapies
- To determine whether a strategy of revascularisation by PCI alters the incidence of sustained ventricular arrhythmia

Inclusion Criteria

All patients enrolled in the REVIVED-BCIS2 trial will be eligible if cardiac implantable electronic device (CIED) interrogation data are available and/or aborted sudden cardiac death status is known. A secondary analysis will be undertaken on patients who had a device preceeding or within 90 days of randomisation; this was a pre-specified analysis within the main trial statistical analysis plan. Inclusion criteria for specific analyses are detailed in the relevant sections below.

Data Collection

Period: from randomisation to death, withdrawal from trial or the end of overall trial follow-up (31/03/2022).

This will include:

Whole cohort
- CIED present? Y/N
- Death? Y/N
  - If Y - Date and cause of death

Device cohort
- CIED type (PPM, ICD, CRT-D, CRT-P)
- Date of insertion
  - Including date of device upgrade if applicable
- Indication (Primary/Secondary prevention)
- All device checks will be reviewed (including local hospital and home monitoring checks) and the following data documented
  - Any reported ventricular arrhythmias Y/N
  - Any ventricular arrhythmia >15 seconds Y/N
    - Type – VT/VF
    - Cycle length, rate, duration, morphology, coupling interval
  - Therapies (CRT-D/ICD only)

        - Type (ATP, ATP followed by shock)
        - Appropriate/inappropriate
        - Number of therapies
        - Success/failure of each therapy
    - Ventricular pacing percentage
    - If ectopy reported - percentage burden
- Medications prescribed at baseline and during follow up
- Anti-arrhythmic medications and indication (ventricular, atrial)
- Ventricular ablation procedures

Other Datasets

The following additional datasets will provide comparative data for analysis

- REVIVED-BCIS2 main trial database and electronic case report forms (eCRF) (London School of Hygiene & Tropical Medicine). The eCRF will be cross checked for reported arrhythmias
- REVIVED-BCIS2 echocardiography core lab database (Guy's and St Thomas' NHS Foundation Trust)
- REVIVED-CMR core lab database (Guy's and St Thomas' NHS Foundation Trust)
- REVIVED-BCIS2 angiography core lab database (University of Glasgow)

DocuSign Envelope ID: B93A3251-5F44-42D1-8DF5-9078C5F17443

Definitions

Aborted sudden death
An appropriate ICD therapy or a resuscitated cardiac arrest

Appropriate ICD therapy
Anti-tachycardia pacing +/- defibrillation for ventricular tachycardia or ventricular fibrillation

Cardiovascular Death
As defined in the main trial and adjudicated by the Clinical Events Committee.

Inappropriate therapy
Anti-tachycardia pacing +/- defibrillation delivered in the absence of a ventricular tachyarrhythmia

Multiple therapies
When multiple therapies have been delivered in in quick succession, any additional appropriate therapy that is >5 minutes after the preceding therapy will be regarded a separate episode of therapy (9). A ventricular arrhythmia storm is defined as >3 separate therapies within a 24 hours period.

Non-sustained ventricular arrhythmia
Any ventricular arrhythmia greater than 3 beats but less than 30 seconds, receiving no therapy

Secondary prevention
ICD placement in a patient with prior sudden cardiac arrest, sustained VT, or syncope confirmed or suspected to be due to ventricular arrhythmia

Sustained ventricular arrhythmia
Ventricular tachycardia >100bpm that lasts for more than 30 seconds **OR** requires termination (e.g. cardioversion) in less than 30 seconds because of hemodynamic compromise **OR** any ventricular fibrillation

DocuSign Envelope ID: B93A3251-5F44-42D1-8DF5-9078C5F17443

Statistical Analysis

Primary composite outcome:

- All-cause death or aborted sudden death

The primary outcome will be assessed in all patients enrolled in the REVIVED trial for whom ICD therapy data are available and/or aborted sudden death status is known. The dataset includes data collected via the main trial eCRF as well as data from the arrhythmia sub-study CRF. The total number of patients eligible for this analysis is estimated to be more than 630 (90%) of the overall 700-patient trial population.

Categorical demographic data will be presented as counts (and percentages) and continuous data as means (and standard deviations (SDs)) or medians (and interquartile ranges) depending on the normality of distribution. The first episode of appropriate ICD therapy will be considered the index event.

All analyses will be by intention to treat according to randomised assignment unless otherwise specified. The primary analysis will be a comparison of the incidence of all-cause death or aborted sudden cardiac death over all available follow-up between the intervention (PCI) and control (OMT) arms of the trial. A time-to-event analysis will be performed on the primary endpoint, with the time to the first event (or censoring) measured from randomisation. The treatment groups will be compared using a Cox proportional hazards model to calculated hazard ratios and 95% confidence intervals. Cox proportional hazards models will also be undertaken adjusted for time since ICD/CRT-D implantation by including ICD/CRT-D implantation as a time-varying covariate. Similar adjustments will be made for the secondary outcomes, but using CIED implantation rather than ICD/CRT-D.

A p-value for the treatment difference will be calculated using a likelihood ratio test. The proportionality assumption underlying the Cox model will be assessed via Nelson-Aalen plots by treatment group and by a more formal test of the Schoenfeld residuals. If there is clear non-proportionality, comparisons will also be made in early and later follow-up using cut-points at 6 months, 1 year, and 2 years. Cumulative event rates will be calculated and presented using Kaplan-Meier time-to-event curves. As a measure of absolute treatment difference, cumulative event rates based on Kaplan-Meier estimates will be compared at 2 years and a 95% confidence interval for the difference calculated. Losses to follow-up are known to be minimal and patients will be included up until the time they experience the event or are censored.

Secondary outcomes:

1. Cardiovascular death, appropriate ICD therapy or sustained VA (time to event)
2. Appropriate ICD therapies or sustained VA (time to event)
3. Appropriate ICD therapies (total number of therapies)
4. Days alive and free from ICD therapy
5. Quality of life assessed by KCCQ scores and EQ5D5L index
6. Health economic analysis

Summary of Outcomes and populations:

Outcome	Analysis	Population
Primary outcome - Death or aborted sudden death	Time to first event	All patients in whom ICD interrogation data and/or aborted sudden death status are known
CV Death, appropriate therapy or sustained VA	Time to first event	All patients in whom CIED interrogation data and/or aborted sudden death status are known
Appropriate ICD therapies	Total number of events	All patients in whom ICD interrogation data are available
Days free from ICD therapy	Difference in means	All patients in whom ICD interrogation data are available
Quality of life	Comparison of means via linear mixed effects model	All patients in whom ICD interrogation data and/or aborted sudden death status are known

Subgroup analyses of the primary outcome

We plan to undertake a limited number of subgroup analyses for the primary outcome. Since the subgroup analyses are secondary analyses and exploratory in nature, the trial has not been powered for these. The pre-specified subgroup analysis will be performed on the following variables using Cox proportional hazards model incorporating tests of interaction:

- CRT v non-CRT device
- Degree of LV dysfunction (stratified by median LVEF)
- Change in LV function (LVEF improved v unchanged/deteriorated)
- BCIS Jeopardy Score (stratified by median score)
- Indication for ICD implantation (primary prevention versus secondary prevention)
- NYHA functional class (0 to 1 vs. 2 to 4)
- Proportion of patients receiving a device by centre (stratified by median proportion)

DocuSign Envelope ID: B93A3251-5F44-42D1-8DF5-9078C5F17443

Power calculation

It is estimated that 40% of this cohort will experience a primary outcome over the entire duration of follow-up (median 3.4 years), which equates to approximately 250 events. Hence it is expected that we will have at least 90% power to detect a hazard ratio of 0.65 or at least 80% power to detect a hazard ratio of 0.70.

		**Power**		
		80%	85%	90%
**Hazard ratio**	0.65	172	196	**230**
	0.70	**249**	285	333
	0.75	382	436	511

DocuSign Envelope ID: B93A3251-5F44-42D1-8DF5-9078C5F17443

References

1. Perera D, Clayton T, Petrie MC, Greenwood JP, O'Kane PD, Evans R, et al. Percutaneous Revascularization for Ischemic Ventricular Dysfunction: Rationale and Design of the REVIVED-BCIS2 Trial: Percutaneous Coronary Intervention for Ischemic Cardiomyopathy. JACC Heart Fail. 2018;6(6):517-26.
2. Gräni C, Benz DC, Gupta S, Windecker S, Kwong RY. Sudden Cardiac Death in Ischemic Heart Disease: From Imaging Arrhythmogenic Substrate to Guiding Therapies. JACC Cardiovasc Imaging. 2020;13(10):2223-38.
3. Ryan M, Morgan H, Petrie MC, Perera D. Coronary revascularisation in patients with ischaemic cardiomyopathy. Heart. 2021.
4. NICE. Implantable cardioverter defibrillators and cardiac resynchronisation therapy for arrhythmias and heart failure: National Institute for Health and Care Excellence; 2014 [Available from: https://www.nice.org.uk/guidance/ta314.
5. Canty JM, Suzuki G, Banas MD, Verheyen F, Borgers M, Fallavollita JA. Hibernating myocardium: chronically adapted to ischemia but vulnerable to sudden death. Circ Res. 2004;94(8):1142-9.
6. Bigger JT. Prophylactic use of implanted cardiac defibrillators in patients at high risk for ventricular arrhythmias after coronary-artery bypass graft surgery. Coronary Artery Bypass Graft (CABG) Patch Trial Investigators. N Engl J Med. 1997;337(22):1569-75.
7. Carson P, Wertheimer J, Miller A, O'Connor CM, Pina IL, Selzman C, et al. The STICH trial (Surgical Treatment for Ischemic Heart Failure): mode-of-death results. JACC Heart Fail. 2013;1(5):400-8.
8. Priori SG, Blomström-Lundqvist C. 2015 European Society of Cardiology Guidelines for the management of patients with ventricular arrhythmias and the prevention of sudden cardiac death summarized by co-chairs. Eur Heart J. 2015;36(41):2757-9.
9. Chen Z, Sohal M, Voigt T, Sammut E, Tobon-Gomez C, Child N, et al. Myocardial tissue characterization by cardiac magnetic resonance imaging using T1 mapping predicts ventricular arrhythmia in ischemic and non-ischemic cardiomyopathy patients with implantable cardioverter-defibrillators. Heart Rhythm. 2015;12(4):792-801.
